# Development of a Computer-Aided Design and Finite Element Analysis Combined Method for Affordable Spine Surgical Navigation With 3D-Printed Customized Template

**DOI:** 10.3389/fsurg.2020.583386

**Published:** 2021-01-25

**Authors:** Peter Endre Eltes, Marton Bartos, Benjamin Hajnal, Agoston Jakab Pokorni, Laszlo Kiss, Damien Lacroix, Peter Pal Varga, Aron Lazary

**Affiliations:** ^1^National Center for Spinal Disorders, Buda Health Center, Budapest, Hungary; ^2^In Silico Biomechanics Laboratory, National Center for Spinal Disorders, Buda Health Center, Budapest, Hungary; ^3^School of Ph.D. Studies, Semmelweis University, Budapest, Hungary; ^4^Do3D Innovations Ltd., Budapest, Hungary; ^5^Department of Mechanical Engineering, INSIGNEO Institute for In Silico Medicine, The University of Sheffield, Sheffield, United Kingdom; ^6^Department of Spinal Surgery, Semmelweis University, Budapest, Hungary

**Keywords:** 3D printing, computed tomography, navigation, finite element simulation, spine surgery, surgical guidance/navigation

## Abstract

**Introduction:** Revision surgery of a previous lumbosacral non-union is highly challenging, especially in case of complications, such as a broken screw at the first sacral level (S1). Here, we propose the implementation of a new method based on the CT scan of a clinical case using 3D reconstruction, combined with finite element analysis (FEA), computer-assisted design (CAD), and 3D-printing technology to provide accurate surgical navigation to aid the surgeon in performing the optimal surgical technique by inserting a pedicle screw at the S1 level.

**Materials and Methods:** A step-by-step approach was developed and performed as follows: (1) Quantitative CT based patient-specific FE model of the sacrum was created. (2) The CAD model of the pedicle screw was inserted into the sacrum model in a bicortical convergent and a monocortical divergent position, by overcoming the geometrical difficulty caused by the broken screw. (3) Static FEAs (Abaqus, Dassault Systemes) were performed using 500 N tensile load applied to the screw head. (4) A template with two screw guiding structures for the sacrum was designed and manufactured using CAD design and 3D-printing technologies, and investment casting. (5) The proposed surgical technique was performed on the patient-specific physical model created with the FDM printing technology. The patient-specific model was CT scanned and a comparison with the virtual plan was performed to evaluate the template accuracy

**Results:** FEA results proved that the modified bicortical convergent insertion is stiffer (6,617.23 N/mm) compared to monocortical divergent placement (2,989.07 N/mm). The final template was created via investment casting from cobalt-chrome. The template design concept was shown to be accurate (grade A, Gertzbein-Robbins scale) based on the comparison of the simulated surgery using the patient-specific physical model and the 3D virtual surgical plan.

**Conclusion:** Compared to the conventional surgical navigation techniques, the presented method allows the consideration of the patient-specific biomechanical parameters; is more affordable, and the intraoperative X-ray exposure can be reduced. This new patient- and condition-specific approach may be widely used in revision spine surgeries or in challenging primary cases after its further clinical validation.

## Introduction

Spinal fixation is a routine procedure for the treatment of unstable spine due to trauma, congenital malformations, degenerative diseases, and tumors (1). The accurate placement of screws in the spine is challenging, given the risk of damage to neighboring anatomical structures (spinal cord, nerve roots, arteries, and veins) (2, 3). Computer-assisted surgery (CAS) has been adopted as a safe and accurate guiding system for the placement of pedicle and lateral mass screws in the spine (4). CAS navigation systems use optical tracking via infrared cameras incorporating 3D geometries from pre-operative CT scans or in combination with fluoroscopy-based imaging (5, 6) or intraoperative CT scans (7). Optimal registration of the spine geometry to the navigational instruments is crucial for precise screw insertion. During surgery, it is often required to perform intraoperative CT scans or use fluoroscopy to re-register the system (5–7). Surgical manipulation after obtaining the intraoperative CT or fluoroscopy images may cause CAS registration errors, which can result in screw malposition. This phenomenon cannot be completely excluded even with a state of the art intraoperative CT technology (7). First concept of individual templates was first introduced by Radermacher et al. (8) in the early 90's by using computer controlled milling device for the manufacturing process. Currently, the 3D-printed patient-specific surgical navigation templates are accurate (9, 10), decrease surgical time, reduce intraoperative X-ray exposure (11), and can be more accessible compared to traditional CT or fluoroscopy-based systems (12, 13). The decline in the costs of 3D-printing technology is expected to continue due to its continuous and fast development (14–16). The MySpine (Medacta International SA, Castel San Pietro, CH) patient- matched pedicle targeting guide for pedicle screw placement (17) is an already clinically available device for the large international spine surgical community. However, in less developed areas of the world, where complex spinal deformity is relatively common and advanced CAS technology is not available (11, 18) 3D-printed templates are still not as widely implemented in the clinical practice, as it would be desirable.

The revision surgery of a lumbosacral non-union can be complicated by an implant related failure, with a broken pedicle screw. In the S1 segment, the convergent bicortical screw trajectory provides superior anchoring compared to any other directions, but the proper insertion of the new screws in a revision surgery due to the broken screw is extremely difficult without surgical navigation. Here, we present a complex clinical case in which the accurate surgical technique required the development of a computer-aided design (CAD) and finite-element analysis (FEA) combined method for affordable spine surgical navigation with a 3D-printed customized navigation template.

## Methods

### Clinical Case

**Figure 1:**
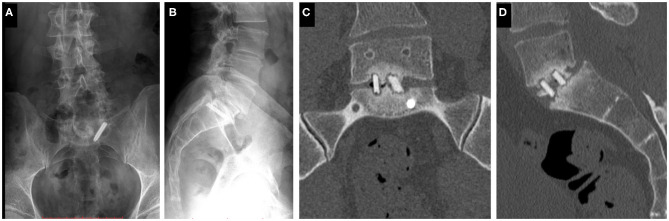
Clinical case of a 38-years-old male patient suffering from low back pain. The patient previously underwent multiple surgeries at the L5–S1 level. A broken left sacral screw can be identified on the standing X-ray images of the patient (**A**. Coronal, **B**. Sagittal plane). Signs of non-union are identifiable in the intervertebral space on the CT scan images of the L5 vertebra and the sacrum (**C**. Coronal, **D**. Sagittal plane).

The study was approved by the National Ethics Committee of Hungary and the National Institute of Pharmacy and Nutrition (reference number: OGYÉI/163-4/2019). Informed consent was obtained from the patient. A 38-years-old patient underwent multiple spine surgeries at the L5–S1 level over a 5-years period with transforaminal interbody fusion (TLIF). During the latest surgery, implant removal and S1 left side nerve root decompression were performed. Six months later, the patient was referred to our institution due to manifestation of mechanical low back pain, with no sign of sensorimotor deficit. Medical imaging at admission (Figure 1) demonstrated a broken S1 left side pedicle screw deep in the sacral bone, and a non-union in the L5–S1 intervertebral space. A revision surgery aiming at the re-fusion of the LV/SI segment was decided.

### Patient-Specific 3D Geometry Definition

**Figure 2:**
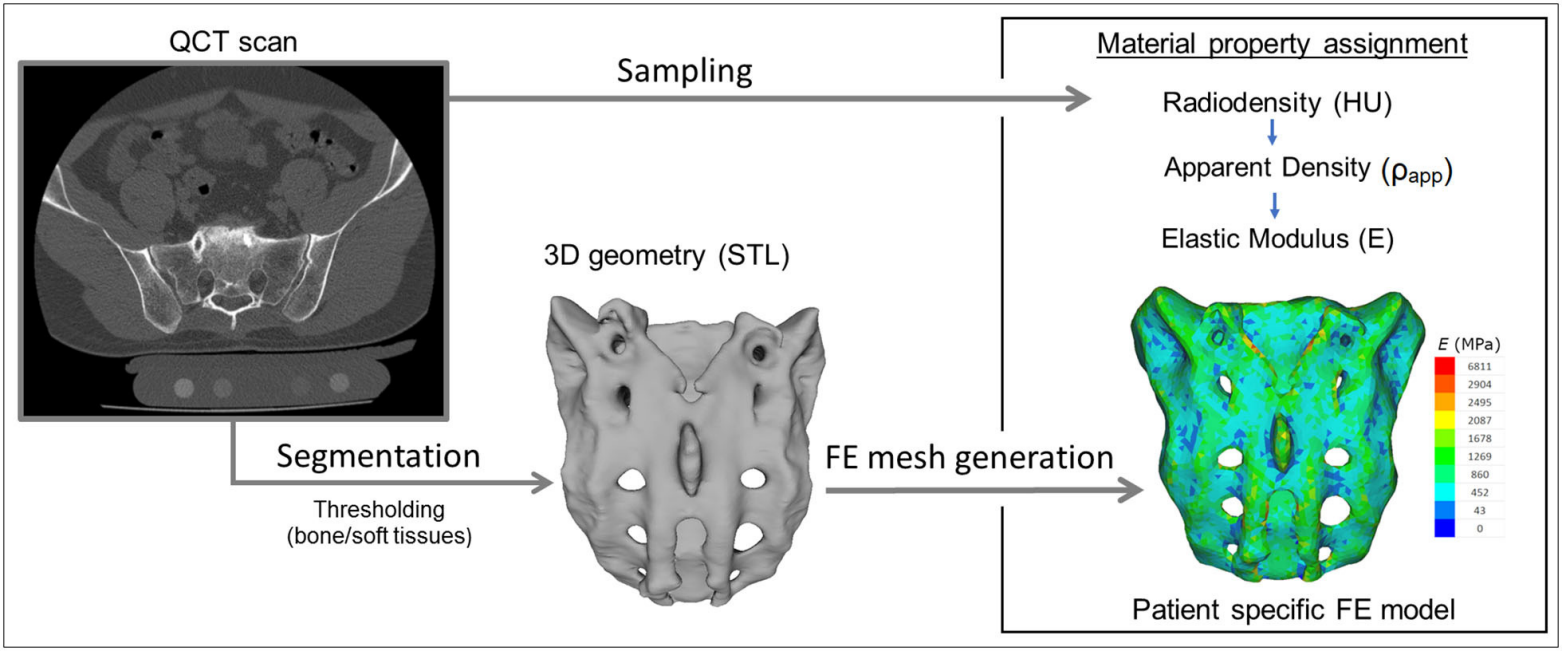
Patient-specific geometry and FE model definition. QCT based segmentation was used to define the sacrum geometry. Hounsfield Unit (HU) values of the QCT images were converted into bone mineral density (BMD) equivalent values. Elastic properties of the sacral bone were estimated using a set of density to elasticity relationships from the literature to convert the BMD equivalent value at each element of the FE mesh to Apparent Density (ρ_app_) (22, 23) and then to the Elastic Modulus (E).

For the study Quantitative Computed Tomography (QCT) scans were used, performed with a Hitachi Presto CT machine (Hitachi Presto, Hitachi Medical Corporation, Tokyo, Japan) using an in-line calibration phantom with five cylindrical insertions of known mean equivalent bone mineral density (BMD) values (0, 0.5, 0.1, 0.15, and 0.2 g/cm^3^) with an intensity of 225 mA and voltage of 120 kV. The imaging protocol was previously defined in the MySpine project (ICT-2009.5.3 VPH, Project ID: 269909) (19, 20), and the images were reconstructed with a voxel size of 0.6 × 0.6 × 0.6 mm^3^. The data were extracted from the hospital PACS in DICOM file format. To comply with the ethical approval of the patient data protection, deidentification of the DICOM data was performed using the freely available Clinical Trial Processor software (Radiological Society of North America, https://www.rsna.org/ctp.aspx) (21). The thresholding algorithm and manual segmentation tools (erase, paint, fill, etc.) in Mimics image analysis software (Mimics Research, Mimics Innovation Suite v21.0, Materialize, Leuven, Belgium) were used (Figure 2) to define the geometry of the sacrum and the broken screw.

The resulting masks (group of voxels) were homogenously filled by preserving the outer contour of the geometrical border in 2D. From the mask, a triangulated surface mesh was automatically generated. On the 3D geometries surface smoothing (iteration: 6, smooth factor: 0.7, with shrinkage compensation) and uniform remeshing was applied (target triangle edge length 0.6 mm, sharp edge preservation, sharp edge angle 60°).

### Surgical Planning and FE Model Generation

**Figure 3:**
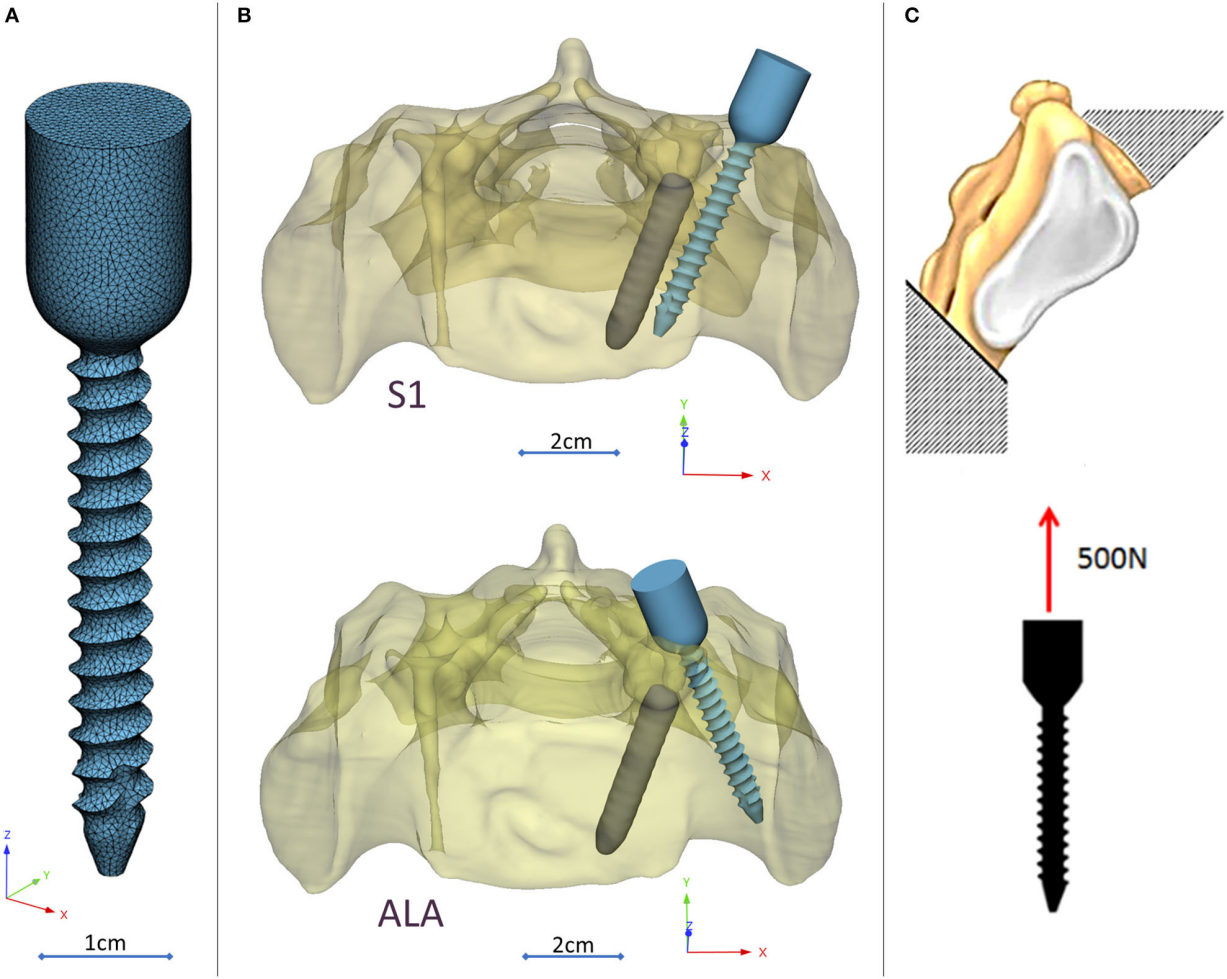
Virtual pedicle screw insertion into the patient-specific sacrum model. **(A)** Modified (monoaxial) virtual model of the pedicle screw. **(B)** Pedicle screw insertion in the convergent position (S1) and divergent position (ALA), the geometrical difficulty caused by the broken screw was overcome in both insertions. **(C)** Boundary condition of the FEA, the sacrum was fixed on the S1 endplate and the caudal 1/3 of the sacrum, 500 N tensile load was applied on the screw head.

A CD Horizon Legacy (Medtronic) polyaxial pedicle screw, 45 mm long and 6.5 mm in diameter, was scanned with the ScanBox 3D scanner (Smart Optics Sensortechnik GmbH, Bochum, Germany). The model of the screw was reconstructed and modified (from polyaxial to monoaxial head) in 3-matic (Mimics Research, Mimics Innovation Suite v21.0, Materialize, Leuven, Belgium) software. The triangulated surface mesh of the screw model was uniformly re-meshed (target triangle edge length: 0.6 mm, sharp edge preservation, sharp edge angle: 60°) (Figure 3A). The screw model was virtually inserted into the 3D model of the patient's sacrum in two positions (convergent: S1, divergent: ALA), using the Mimics software's STL import tool (Figure 3B) with the consideration of the broken screw. Two non-manifold assemblies were created in the Mimics software containing the broken screw, implanted screw, and sacrum for the convergent (S1) and divergent (ALA) positions.

The assembly was exported to the 3-matic software where nine FE meshes were generated for each of the implantation scenarios (S1, ALA). The broken screw, inserted implant, and sacrum-implant interface had a triangle set with an edge length of 0.6 mm. The outer surface of the sacrum mesh was changed in the nine models by defining the uniform triangle mesh edge length as 2.0, 2.5, 3.0, 3.5, 4.0, 4.5, 5.0, 5.5, and 6.0 mm. Adaptive meshing protocol was used for the volume mesh creation with 10-node tetrahedral elements. The maximum edge length of the meshing process corresponded with the initial edge length of the sacrum surface mesh (Supplementary Figure 1), for the screw and the broken screw the same FE mesh parameters was used in all models.

The material property assignment for the volumetric elements representing the sacral bone tissue was performed in two steps (Figure 2): first, conversion of the HU (*Hounsfield Unit*) values to BMD values based on the in-line phantom was performed, the conversion curve was assumed to be linear according to studies (22, 24). The obtained relationship between the HU and the apparent bone density for each element was ρ_app_ = −0.0829 + 0.0026 *HU* (ρ_app_ [g/cm3]). Then, the bone tissue was assumed to be isotropic and linearly elastic with a Poisson's ratio of 0.3 (25). Conversion curves between the density and the elastic modulus of the bone were based on the correlation established by Kopperdahl et al. (23), *E* = −34.7 + 3,230·ρ_app_, (bone elastic modulus = E [MPa]). The FE models were exported to the Abaqus/CAEv11 (Dassault Systemes, Simulia Corp, Providence, RI, USA). For the broken and the inserted pedicle screws the material properties were defined as follows: Poisson's ratio of 0.3 (26), elastic modulus of 114,000 MPa (26). Between the screws and the sacrum tie connections were used. The finite element model was subjected to a static of 500 N tensile load applied to the screw head and it was fixed at the S1 endplate and lower third of the sacrum (Figure 3C).

### Navigation Template Design, Manufacturing, and Accuracy Evaluation

**Figure 4:**
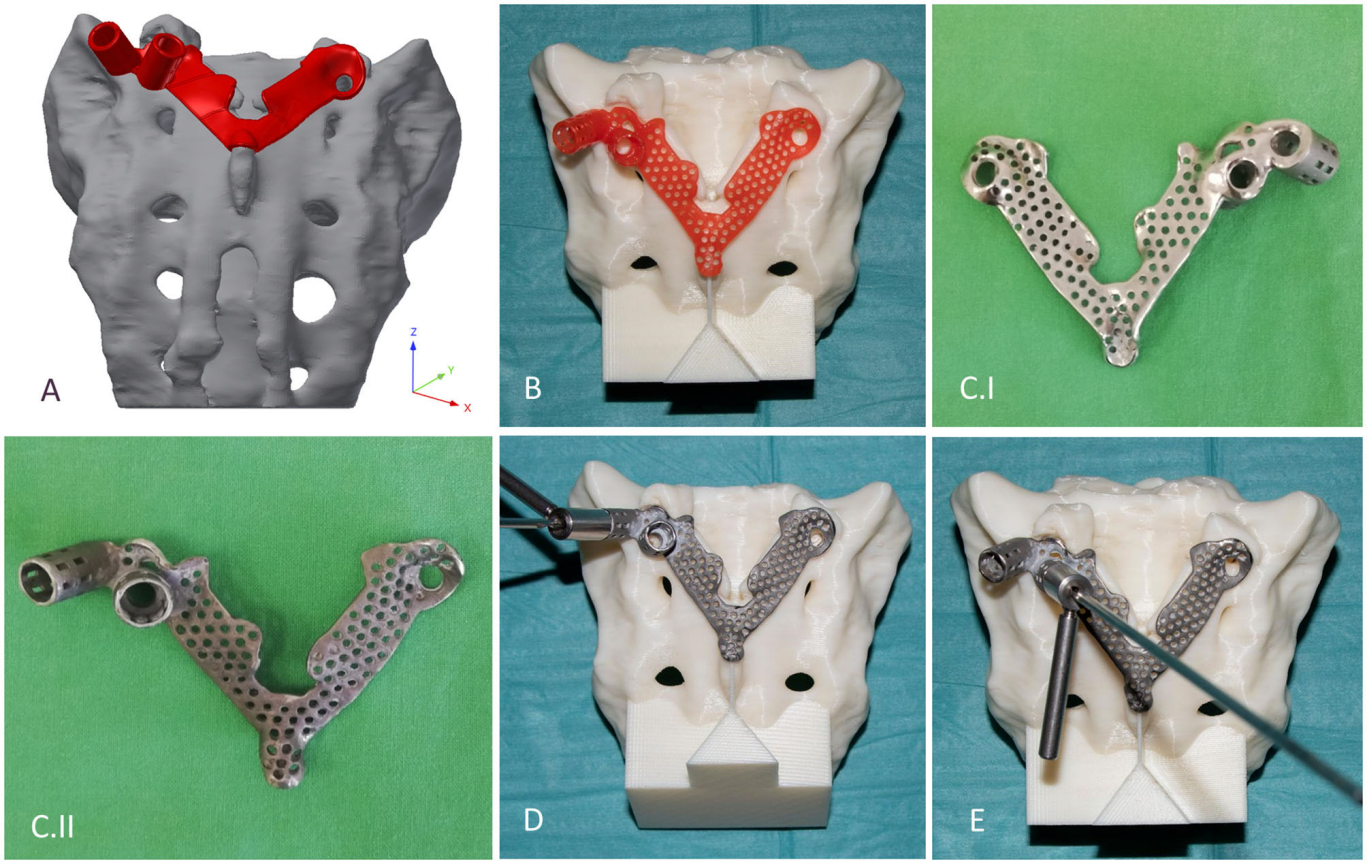
Design, manufacturing, and accuracy evaluation of the navigation template. **(A)** Template's virtual model created via CAD software. **(B)** 3D-printed (MSLA technology) template (red) fits exactly on the 3D-printed (FDM technology) patient-specific physical model. **(C.I,II)** Final navigation template created via investment casting from cobalt-chrome (**C.I** ventral surface polished, **C.II** dorsal surface). Evaluation of the drilling accuracy was performed on the physical model in the **(D)** convergent position (S1) and **(E)** divergent position (ALA).

**Figure 5:**
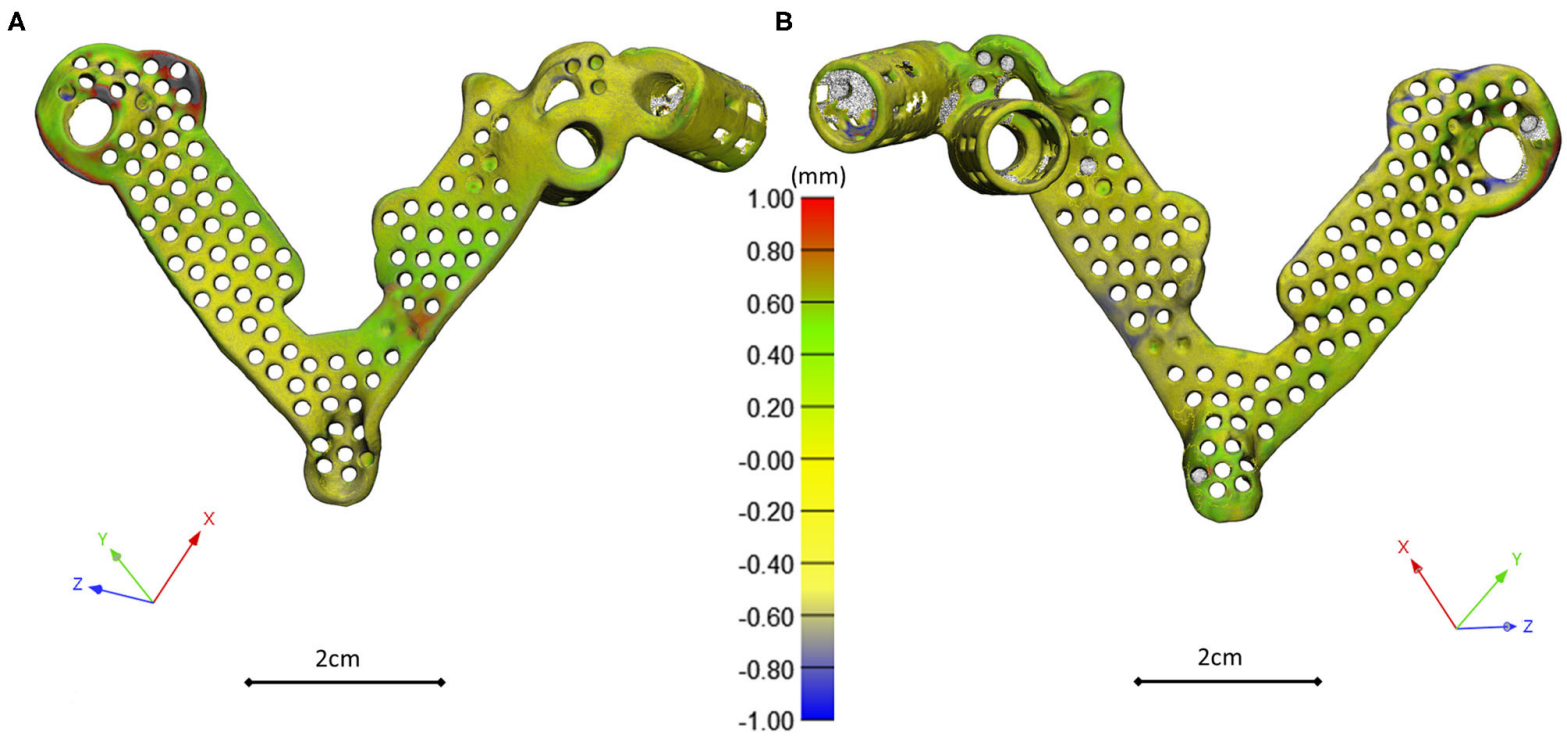
3D scanning based geometrical accuracy measurement. Cobalt-chrome investment casted navigation template's geometrical accuracy compared to the 3D-printed navigation template model created with MSLA technology. The color map (Scale; min = −1 mm, max = 1 mm) shows the geometrical difference, projected on the 3D-printed navigation template triangle based mesh model vertices (**A** ventral view, **B** dorsal view).

The template design was based on the axis of the virtually inserted screw, individual geometry, and surface of the cranial/dorsal part of the sacrum. In the 3-matic software the two axes and surface for the template/sacrum contact were defined based on the STL assembly (broken screw, inserted implant, sacrum). The contact surface and the axes were exported to the Autodesk Fusion 360 (Autodesk Inc., California, U.S.A.) CAD software which was used for the finalization of the design (Figure 4A). The virtual model of the template was printed with masked stereolithography (MSLA) technology based 3D-printing machine (VOXEL L 3D-Printer; Parameters: building size: 125 × 65 × 65 mm, layer thickness: 0.05 mm; Material: Voxeltek Cast Resin; Do3D, Hungary) (Figure 4B). The used photopolymer resin can be used as a pattern for investment casting. Finally, the model was produced in a dental laboratory via investment casting (Hexacast induction centrifugal casting machine; Parameters: start torque: 0–21 Nm, maximum melting mass: 100 g, max heating: 1,750°C, dimensions (width × height × depth): 660 × 390 × 645 mm; Material: CoCr; PiDental, Hungary) from cobalt*-*chrome (Figures 4C.I,II). The accuracy of the casted part was tested via 3D scanning ScanBox 3D scanner (Smart Optics Sensortechnik GmbH, Bochum, Germany) and compared to the 3D-printed model. The point clouds resulting from the scanning were aligned and compared in the 3-matic software with the part comparison module (Figure 5).

The accuracy of the template was tested on a patient-specific sacrum physical model, 3D-printed with a Fused Deposition Modeling (FDM) printer (Dimension 1200es 3D-Printer; Parameters: building size: 254 × 254 × 305 mm, layer thickness: 0.330–0.254 mm; Material: ABSplus/ivory; Stratasys, Israel). The drill template was placed on the FDM sacrum model; then, a cylinder inlet was connected to the template to support the drill bit, and the drilling of the model was performed according to the S1 and ALA positions (Figures 4D,E).

The template was removed and two CT scans were performed of the sacrum model with drill bits inserted in the S1 and ALA positions. The CT scan images were imported into the Mimics software where the segmentation (thresholding) and 3D reconstruction of the patient-specific FDM sacrum model geometry and drill bits were performed. The models were registered to the initial sacrum geometry derived from the QCT via point based rigid registration by selecting anatomical landmarks in the caudal part of the sacrum (Supplementary Figure 2). This step was followed by an automatic global registration inside the 3-matic software. The registration accuracy was measured with the part comparison module of the 3-matic software (Supplementary Figure 3). The centerline for the drill bit 3D geometry was defined and an analytical primitive (cylinder, 2.5 mm in diameter) was fitted to define the drilling axis. In 3-matic software 3D angle measurement tool, line to line module (World Coordinate System, XYZ coordinates) was used to quantify the accuracy of the screw insertion by defining the angels in the 3D space between the virtual screw centerline and the drill bit centerline.

## Results

### Navigation Template Geometrical Accuracy and Performance

**Figure 6:**
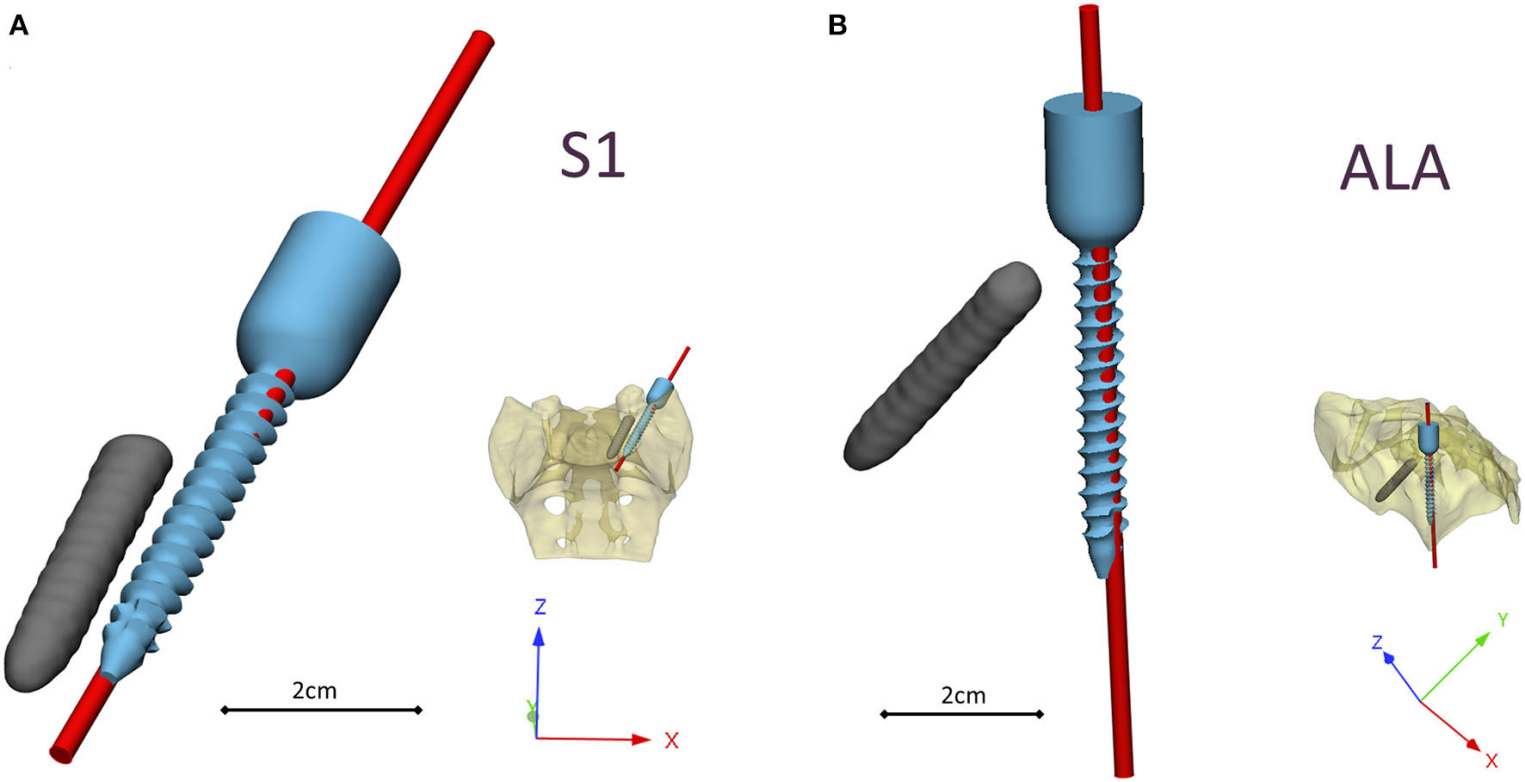
Visualization of the navigation template compared to the virtual plan. The red cylinders represent the drill bits' axes in the **(A)** convergent position (S1) and **(B)** divergent position (ALA), based on the evaluation performed on the patient-specific physical model. The broken screw and implanted screw geometry are part of the virtual surgical plan based on the patient's QCT.

The investment casted cobalt-chrome drill template retains the geometrical properties of the pattern (3D-printed drill template model created with MSLA technology) based on the 3D scanning evaluation Figure 6. To evaluate the drill template's performance, we used a 3D-printed patient-specific physical model. The physical model with the two drilling positions was scanned with CT, segmented and aligned to the virtual surgical plan (Supplementary Figures 2, 3). The drill template allowed a highly accurate screw insertion in both investigated positions (Figure 6). The cylinders representing the drilling axes were not perfectly colinear and coincident with the screws in the virtual surgical plan, the 3D angle between the screw centerline and the drill bit centerline for the S1 was α = 4.42° and for the ALA was α = 2.4°.

### FEA Results

**Figure 7:**
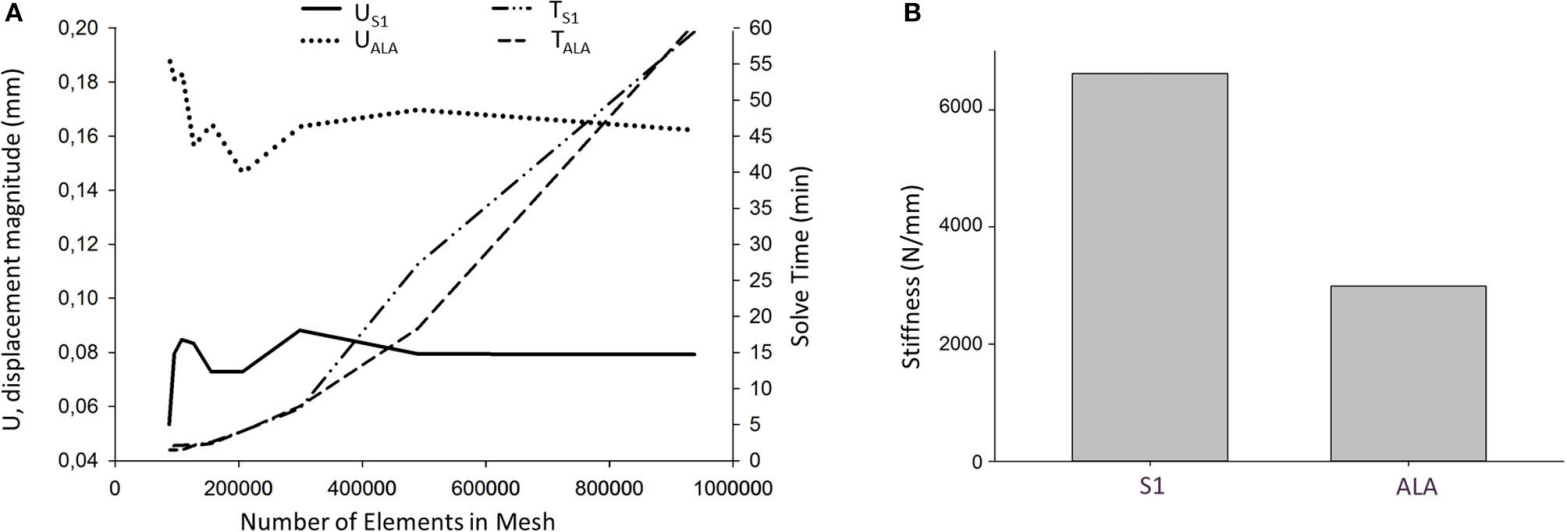
FE simulation results. **(A)** Convergence analysis for the average U, displacement magnitude (nodes of the middle 1/3 of the screw head) in convergent (U_S1_) and divergent (U_ALA_) screw positions at different mesh element numbers. Solve time distribution (right) at different mesh element numbers [convergent (T_S1_) and divergent (T_ALA_) screw positions]. **(B)** The convergent screw insertion (S1) is stiffer compared to the divergent (ALA) insertion.

Nine models were created for each screw insertion scenario (*N* = 9, S1 and *N* = 9, ALA) with increasing element numbers based on the virtual surgical plan. The FE simulation results converged above 2^*^10^5^ elements for both screw insertion scenarios at ~5 min solve times on two cores. The solve time at two cores for the S1 orientation was higher compared to the ALA (Figure 7A). The convergent bicortical screw insertion (S1) provided a stiffer (6,617.23 ± 1,106.24 N/mm) situation based on the nine FE model compared to the monocortical divergent screw position FE model values (2,989.07 ± 240.24 N/mm) (Figure 7B).

## Discussion

Comparative studies have been published in recent years (27–29), demonstrating the reliably, efficacy, and advantages of 3D printed navigational templates compared to other navigational methods or free-hand technique. In this study, we present a technology development process in order to create a patient-specific drill template in a complex clinical case, in which a broken screw causes geometrical difficulty for new screw insertion. In order to safely insert the new screw, without compromising the local bone structure we developed a virtual surgical plan based on the QCT of the patient. This allowed us to test two different screw positions in the model and to design a drill template for safe screw insertion at the level of the first sacral vertebra with a geometrical difficulty caused by a broken screw from a previous surgery. The present study demonstrates the accuracy and applicability of a developed workflow which allows the creation of an affordable, metal, individualized navigational template by integration of FEA in the design and surgical planning process.

The integration of FEA in the pedicle screw intraoperative navigation was investigated by Van den Abbeele et al. (30), however, the application of FEA in the design process of a navigational template in spine surgery by integrating the patient bone mineral density related material properties is new. The results of the simulations showed that the convergent S1 insertion is significantly stiffer than the divergent ALA insertion. This finding is supported by cadaveric experimental studies (31, 32) and clinical experience as well (33). The biomechanical difference of the convergent and divergent insertions rely on the differences in the local bone mineral densities (34).

The combination of the 3D-printing technology and cobalt-chrome casting makes the manufacturing process more affordable. Investment casting of cobalt-chrome is a widely used technology in dental laboratories (35). 3D-printed patterns for casting is an accepted method in dentistry (35, 36); however, its application in spine surgery navigational templates is novel. The production of individualized metal navigational templates for screw insertion can be achieved via selective laser sintering 3D-printing technology of titanium-based alloys (37), but at a higher cost and lower accessibility compared to dental casting. Metal templates are robust, resistant to damage, and can also be easily autoclaved (37).

**Figure 8:**
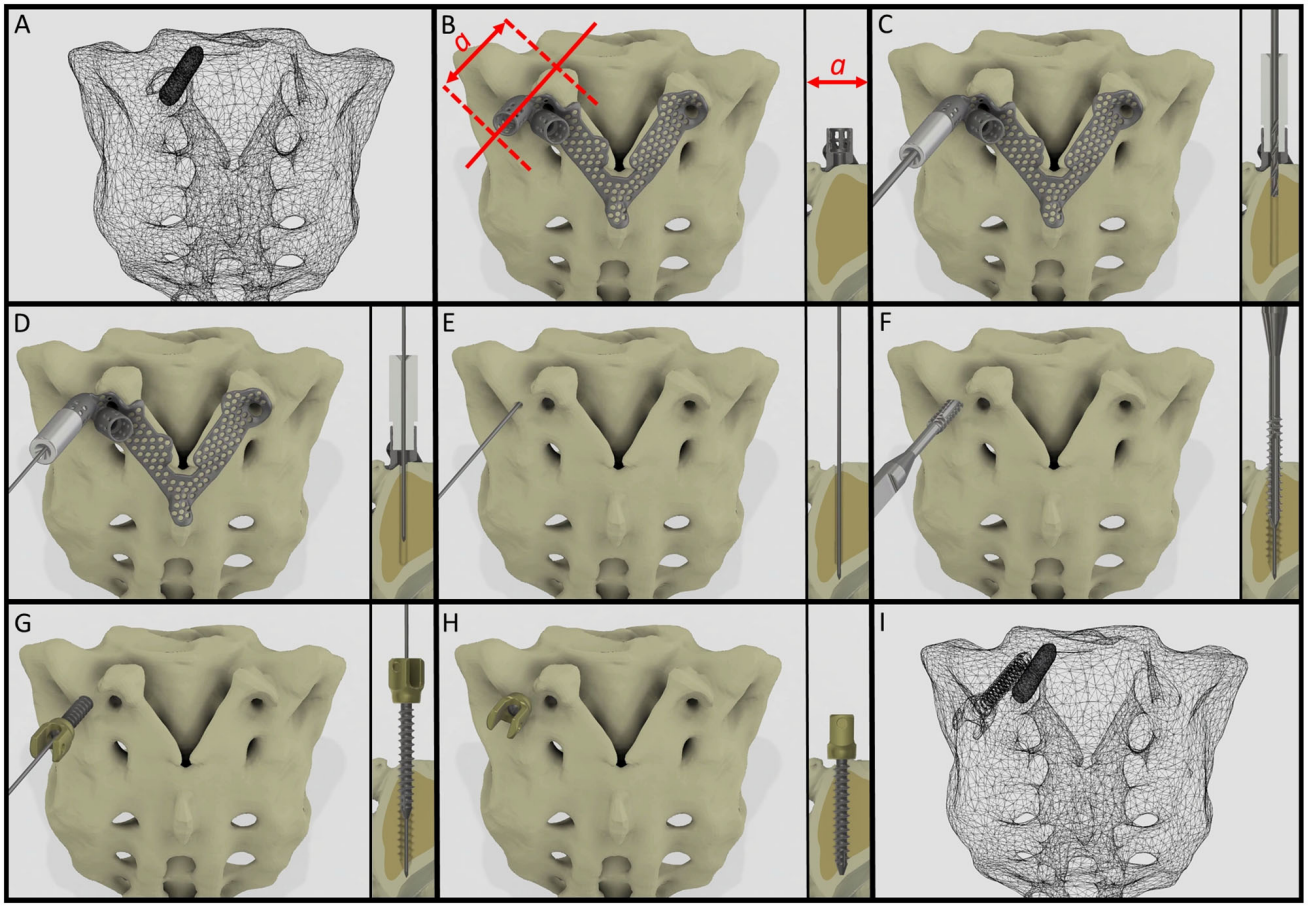
Proposed surgical technique for safe and accurate screw insertion in convergent position. **(A)** Transparent surface mesh of the patient's sacrum with the broken screw. **(B)** Section plane dimension and orientation, and drill template position on the sacrum. **(C)** Stainless steel cylinder inlet connected to the navigation template for the drill bit. **(D)** Stainless steel cylinder inlet connected to the template for the Kirschner wire. **(E)** The inlet cylinder and template are removed, the Kirschner wire's position is unchanged. **(F)** Cannulated tap is introduced along the Kirschner wire. **(G)** Cannulated pedicle screw is introduced in the sacrum along the Kirschner wire. **(H)** Final position of the screw. **(I)** Transparent surface mesh of the sacrum with the broken and convergently inserted pedicle screw geometry.

It is widely accepted in the literature to use cadavers for testing, evaluating the fitting accuracy of a navigational template (38). FDM technology can produce geometrically accurate spine physical models (39) and the different designs can be tested as well as the drilling accuracy can be evaluated. The use of FDM models for design process evaluation and development is advantageous due to the possibility to include retrospective patient imaging data with complex anatomical/geometrical variation (deformities, tumors, etc.) which is extremely difficult to control and integrate in the case of cadaveric specimen studies. Based on our FEA results, the S1 screw insertion's surgical plan and drill template position is recommended for surgical implementation. Despite the fact that the virtual screw axis and the drill bit centerline are not colinear and coincident (3D angle α > 0) according to the Gertzbein-Robbins scale (40) the template theoretically allows an accurate (grade A) screw insertion (Figure 6). We present the surgical technique for the screw insertion with the developed drill template (Figure 8 and Supplementary Video 1). The suggested screw insertion surgical technique uses the philosophy of the minimally invasive pedicle screw insertion techniques (MIS) by using a Kirschner wire, cannulated tap, and pedicle screw. This technique can easily be performed by any spine surgeon familiar with MIS pedicle screw insertion.

Limitations of this study include the fact that the developed template is presented using a single case, however the workflow can be applied for different parts of the spine with different geometrical difficulties/pathologies. The presented FEA models' loading conditions are simplified as well as the material property assignments; more complex FEA investigations would be desirable. In the future, a randomized study of specific subtypes of spinal pathologies (tumors, deformities, etc.) with a larger sample size would be preferred to demonstrate the clinical efficacy and cost-effectiveness of the developed methodology.

## Conclusion

A patient-specific template for pedicle screw insertion allows the surgeon to insert the screw into its optimal position. The advantages of our technique compared to the conventional surgical navigation tools are the affordability, the potential to reduce intraoperative X-ray exposure, and the possibility for the consideration of patient-specific bone geometry and biomechanics. This new patient- and condition-specific approach can be widely used in revision spine surgeries or in challenging primary cases after its further clinical validations.

## Data Availability Statement

The datasets generated for this study are available on request to the corresponding author.

## Ethics Statement

The studies involving human participants were reviewed and approved by National Ethics Committee of Hungary and the National Institute of Pharmacy and Nutrition (reference number: OGYÉI/163-4/2019. The patients/participants provided their written informed consent to participate in this study. Written informed consent was obtained from the individual(s) for the publication of any potentially identifiable images or data included in this article.

## Author Contributions

PEE, AL, PPV, MB, and DL: research design. PEE, AL, MB, BH, AJP, and LK: acquisition of data. PEE, AL, MB, BH, AJP, and DL: analysis and/or interpretation of data. All authors: drafting the paper or revising it critically and approval of the submitted and final versions.

## Conflict of Interest

MB was employed by the company DO3D Innovations Ltd. The remaining authors declare that the research was conducted in the absence of any commercial or financial relationships that could be construed as a potential conflict of interest.
