# Supplementary material for: Development of a Computer-Aided Design and Finite Element Analysis Combined Method for Affordable Spine Surgical Navigation With 3D-Printed Customized Template
Source: Front Surg. 2021 Jan 25;7:583386. doi: 10.3389/fsurg.2020.583386 (PMC7873739; doi:10.3389/fsurg.2020.583386)
Supplement: Supplementary file 1 [file Image_1.pdf]

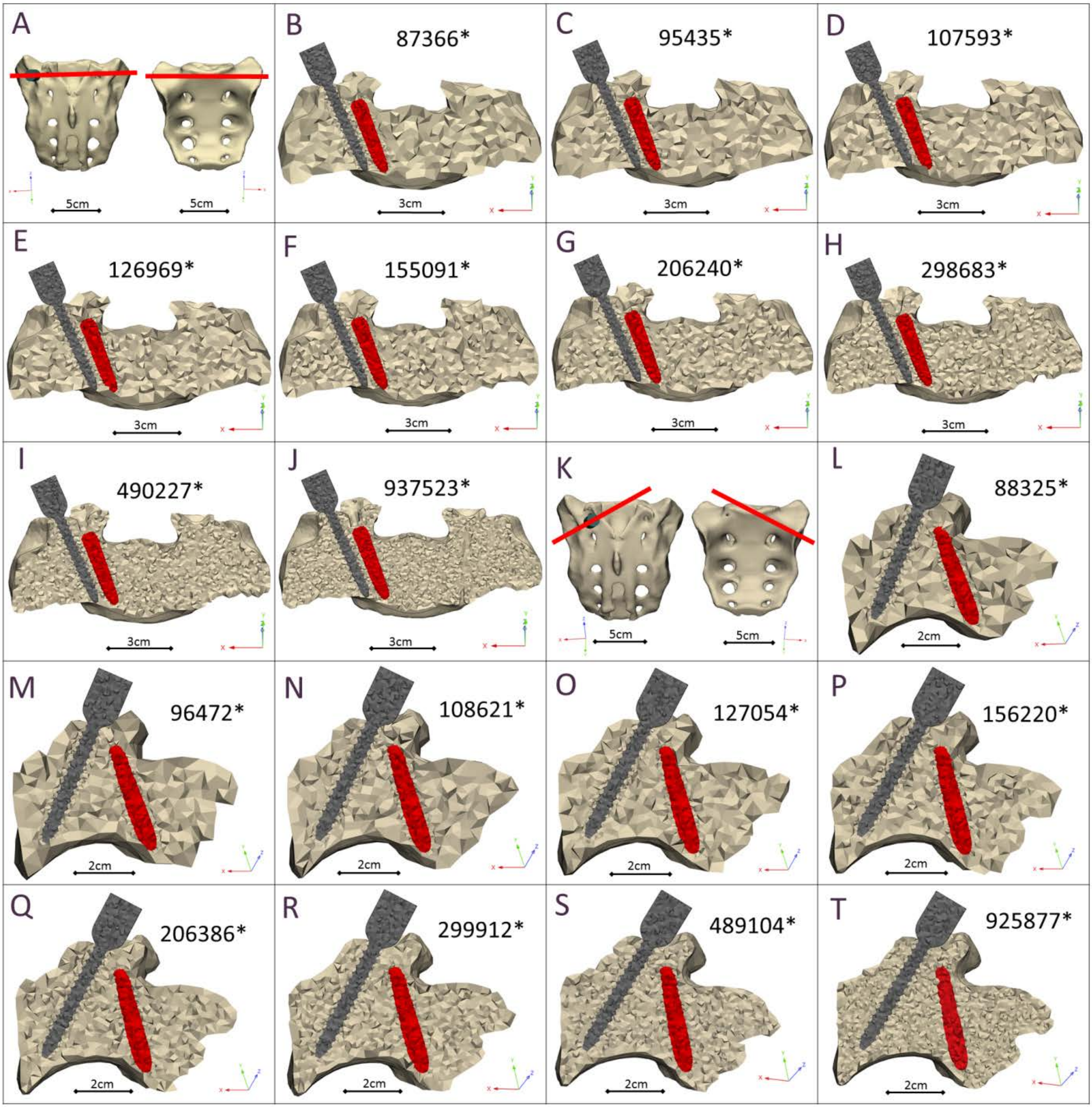

Supplementary Figure 1. Finite element models of the sacrum with convergent (S1) and divergent (ALA) screw insertions. (**A**) section plane in the convergent screw insertion model (posterior and anterior view). (**B**-**J**) the screw is inserted into the convergent position in close proximity to the broken screw (red). (**K**) section plane in the divergent screw insertion model (posterior and anterior view). (**L**-**T**) the screw is inserted into the convergent position in close proximity to the broken screw (red). The FE models' mesh element numbers (*) vary according to mesh density.
