# Supplementary material for: Development of a Computer-Aided Design and Finite Element Analysis Combined Method for Affordable Spine Surgical Navigation With 3D-Printed Customized Template
Source: Front Surg. 2021 Jan 25;7:583386. doi: 10.3389/fsurg.2020.583386 (PMC7873739; doi:10.3389/fsurg.2020.583386)
Supplement: Supplementary file 2 [file Image_2.pdf]

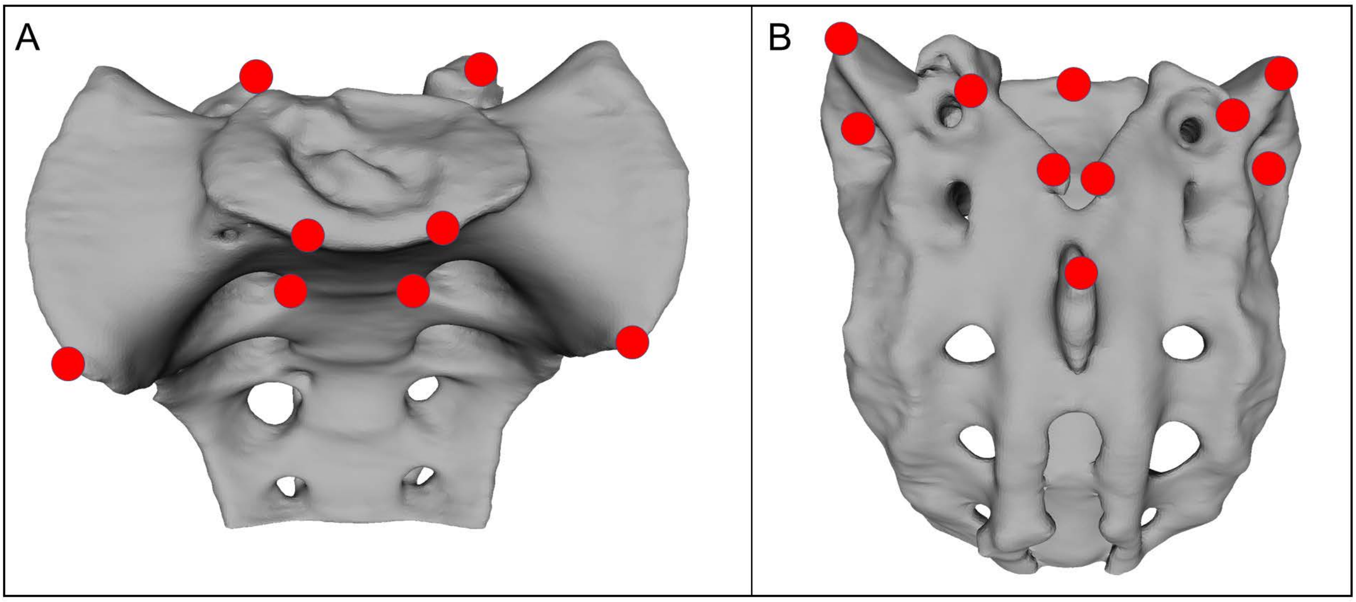

Supplementary Figure 2. Control points selection for rigid surface registration. Eight control points from the superior-ventral (**A**) region and ten control points from the superior-dorsal (**B**) region were selected from the reference (patient QCT) and from the aligned geometry (physical model CT scan) respectively. The red circles represent the registration points selection areas.
