# Supplementary material for: Development of a Computer-Aided Design and Finite Element Analysis Combined Method for Affordable Spine Surgical Navigation With 3D-Printed Customized Template
Source: Front Surg. 2021 Jan 25;7:583386. doi: 10.3389/fsurg.2020.583386 (PMC7873739; doi:10.3389/fsurg.2020.583386)
Supplement: Supplementary file 3 [file Image_3.pdf]

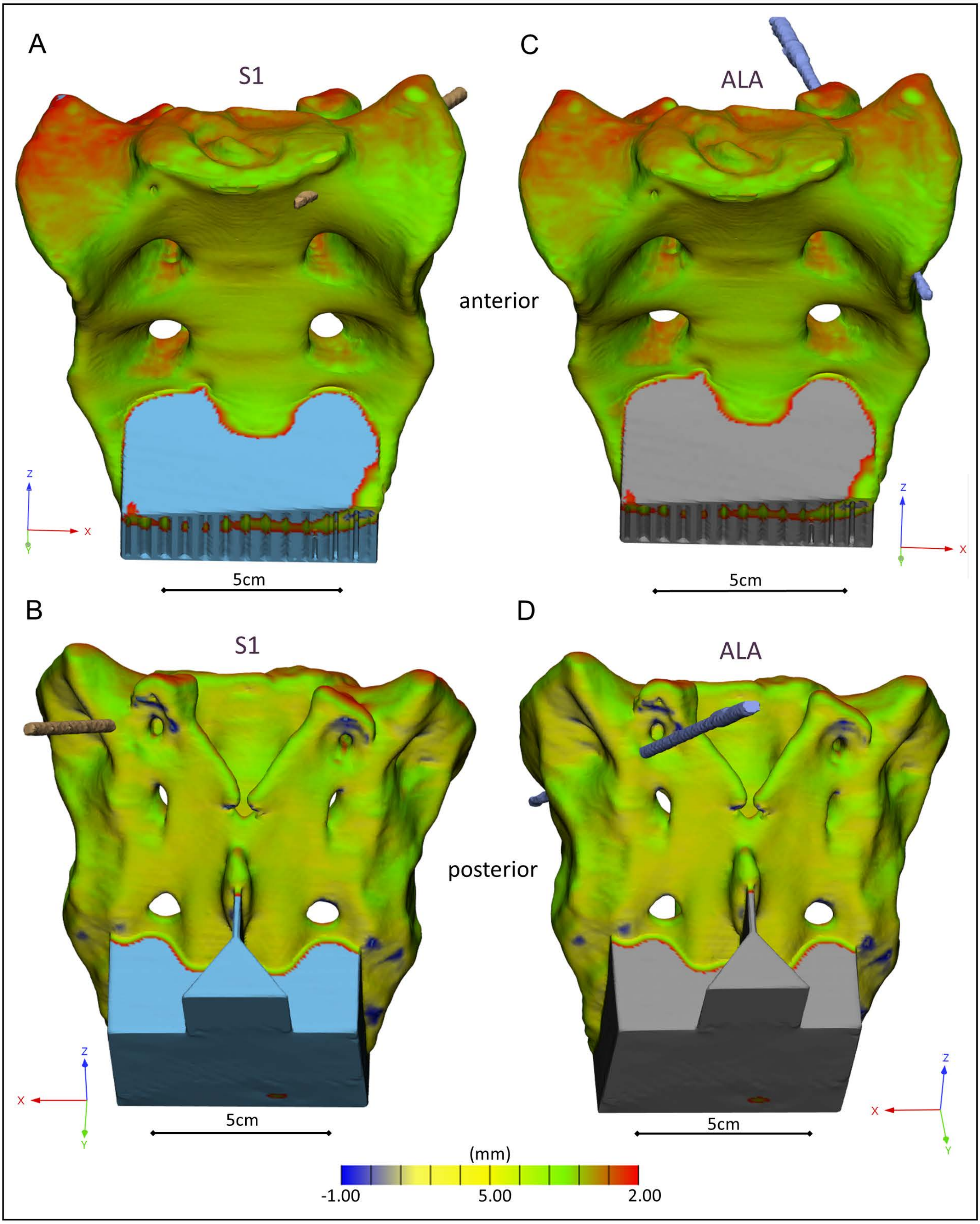

Supplementary Figure 3. Alignment accuracy evaluation of the drilled, patient specific physical model compared to the virtual sacrum model. (**A**, **B**, **C**, **D**) Surface mesh of the patient specific physical model and the drill bit (2.4 mm diameter) in S1 position (**A** , **B**) and ALA position (**C**, **D**) registered (rigid registration, point based + global registration) to the segmented patient sacrum derived from the QCT (**Fig 2**).
